# An Address

**Published:** 1864-03

**Authors:** G. A. Mills


### AN ADDRESS.

Read before the Brooklyn Dental Association.

BY DR. G. A. MILLS.

*Gentlemen of the Brooklyn Dental Association*:—As the subject for our consideration to-night is, what we have gained by meeting together, I have tried in a feeble manner, to give you in a few words, scme of my own experience: I will speak first of what has been gained socially.

Two years ago last September, I came to Brooklyn a stranger and unknown. I knew none of my brethren in the profession, and none of them knew me; none called to see me, and I called to see none; and until I met them in this Association, I did not know their faces, not even my nearest neighbors.

A few weeks previous to my meeting with this Association (I know not why) I had been wishing more than ever that we could have a Dental Association in the city of Brooklyn. My desire began to run high, and I did venture one snowy day, to ring the bell of one of my brother dentists. He received me cordially,—we were both *Yankees*, therefore a little shy. Before I left, I introduced the subject of a Dental Association in Brooklyn. He went on and gave me his experience, and said it was a failure. From this time the subject was dropped, and I returned home and consoled myself that it "was no go." In a few days after I was much gratified on receiving an invitation to meet with this Association, at the office of Dr. Hill. I determined to go—I did not know until this time that such a body was in existence.

I shall never forget my first impressions as I entered

Dr. Hill's office; I said to myself, this is a hairy set. I was welcomed by our good brother, Dr. Atkinson, and the moment I grasped his hand I felt I should have a friend in him, and yet looked on him with not a little curiosity. I then cast my eye across the room and took a "bird's eye view" of our venerable looking Secretary, but did not read him so quickly. I soon compared the length of his head by the length of his hair, and came to the conclusion that there was not much difference, both being long; and thought I should like him better when I knew him more, and such is the fact. I was soon invited to join, and accepted—was duly elected, signed the constitution, and was admitted to its deliberations. There were others there like myself to survey (but not to be caught). The meeting passed away pleasantly, and at the usual time we refreshed the *inner man* over sandwiches, etc., shook hands all round, and adjourned to meet at Dr. Wheeler's in two weeks from that night. I have felt that I was a better man—I now know many of my brethren in the profession, and am known of them.

I have formed a strong attachment to this Association, and have made such friends as *money* could not purchase. There are those *here* that I have learned to love with a love that only death will sever.

So I feel that if I have gained no more, in this I have gained much. We need to associate together, it breaks down all jealousy, it makes larger men of us, it developes our social nature, enlarges our researches, and puts better fillings into the teeth of our patients, more gladness in our own hearts, and more money in our pockets.

We shall be (as we are) better known and appreciated by thousands, shall have better success, more satisfaction, live longer and die happier, and our works will live after we have passed away, and be esteemed by men.

What we have learned in Dental Pathology, which we once looked at as a mountain, and as immovable, is reduced to a mole-hill. How many of us a few months since, supposed that we should be able to puncture flesh and bone to the abscess, and by the introduction of a little piece of broom corn enclosed with cotton saturated with creosote and iodine, we could disperse and literally cure it, beyond a doubt.

But to-night we can testify to a legion of cases that have been successfully treated in this manner, and does it not do us good. In this have we not gained something? Take the use of creosote, we use it more and love it better, the odor is not as disagreeable as formerly. We have been accustomed to keep it (if at all) in hœmeopathic doses, and in some very remote place, now it stands side by side with the cologne bottle, and we need not ask our brother dentist as we meet him in the street, if he is a member of the Brooklyn Dental Association; if he smells of creosote you can set him down as an active member in good and regular standing. In a word, creosote in our practice has worked wonders, and I am not quite certain, but in the next century, statistics will record less, by one-half, the number of mortalities. *Cause, creosote.*

Last, but not least, the mallet in our practice is making its changes. I can say with my whole heart, *God bless him* who introduced it in filling teeth; who has taken up the use of it and persevered, has ever laid it down? I answer, no one, and never will. Its use is *convincing*, and carries conviction with every blow.

I feel that it has marked a new era in my life, my operations are more satisfactory to me than before, I have never put in so good fillings as I have since its use in my hands.

Teeth that I would once have *extracted* or compromised with amalgam, are now filled from the foundation to the end, a solid glistening plug of *gold*, bidding fair to do a good service as long as the teeth remain in the socket. I could say more of the use of the mallet, if time would permit, but will leave it until some future day.

I am told by some that the reason they do not join us is, "they can not afford it." Gentlemen, I can not afford *not*

to be a member; there is no stock so good for me, even if they count dollars and cents—for curiosity I have made an estimate of what the whole cost has been to me since I joined not one year since, I can not get it over $30.00.

Now what I have gained in the treatment of alveolar abscess alone, I have made clear in money (and it is clean money too) $100.00, and by the mallet since last May, I have enhanced my business not one cent less than $1,000.00, and if I were to reckon in my physical improvements I should set my figures high.

I believe fully that I have prolonged my life a good 25 years. Gentlemen, this Association is a largely paying one, financially, physically, socially and morally.

